# Change in self-reported activity in chronic low back pain after 13 years – a prospective longitudinal cohort study in primary healthcare

**DOI:** 10.1186/s12891-025-09371-8

**Published:** 2025-12-03

**Authors:** L. Nordeman, A. Grimby-Ekman, CM Ho-Henriksson, E. Enhörning, M. Hellgren, A. Bergenheim

**Affiliations:** 1 Research, Education, Development, and Innovation, Primary Healthcare, Region Västra Götaland, Sven Eriksonsplatsen 4, Gothenburg, 503 38 Borås Sweden; 2 Department of Health and Rehabilitation, Unit of Physiotherapy, Institute of Neuroscience and Physiology, Sahlgrenska Academy, University of Gothenburg, Gothenburg, Sweden; 3 School of Public Health and Community Medicine, Institute of Medicine, Sahlgrenska Academy, University of Gothenburg, Gothenburg, Sweden; 4 Närhälsan Lidköping Primary Care Rehabilitation center, Region Västra Götaland, Lidköping, Sweden; 5 Närhälsan Uddevalla Primary Care Rehabilitation center, Region Västra Götaland, Uddevalla, Sweden; 6 The Skaraborg Institute Skövde, Skövde, Sweden

**Keywords:** Low back pain, Primary care, Cohort, Women

## Abstract

**Background:**

The knowledge about the improvement of body function, activity and participation in chronic low back pain (CLBP) over a period of over ten years is still insufficient. The study aimed to investigate the long-term change for body function, self-reported activity due to CLBP e.g. Roland Morris Disability Questionnaire (RMDQ) and work participation.

**Methods:**

A 13-year prospective longitudinal cohort study of women with CLBP seeking primary healthcare. Women (*n* = 130) with CLBP (> 12 weeks) were included in 2004 to 2005 (baseline), after two and 13 years. The assessment included questions about socio-demographic data, comorbidity, and pharmacological treatment, physical capacity-tests, and patient reported outcome questionnaires. Changes in measurements, for physical capacity, pain intensity, pain localisations, RMDQ, symtoms of anxiety and depression, clinical stress symptoms, and health-related quality of life, between baseline and the 13-year follow-up were calculated. Depending on the data level and distribution, either parametric or non-parametric tests were applied. A mixed effect model was used to analyse repeated measures of RMDQ from baseline to the two-year and 13-year follow-ups, comparing the group with localised CLBP and CLBP + widespread pain (WP) group. The RMDQ was dependent variable and age, education level, pain intensity, 6-Minute Walk Test, symptoms of stress, and depression were included as confounding factors.

**Results:**

67% (87/130) could be followed up after 13 years. 26% of the participants (22/86) fulfilled the criteria for WP at baseline. All measurements showed small improvements or were stable, except for hand grip strength. The mean change in RMDQ was − 3.3 (4.1) points. 73% (63/86) improved at least one point and 40% (34/86) improved ≥ 5 points on RMDQ from baseline. RMDQ improved in both the groups CLBP and CLBP + WP over the 13-year period (*p* < 0.001) when adjusting for age, education, pain intensity, 6-Minute Walk Test, symptoms of stress, and depression.

**Conclusions:**

All outcome measurements, except for hand grip force either showed small improvements or remained stable over a period of 13 years. For activity related to CLBP, no differences were found between the CLBP group and the CLBP + WP group when adjusting for pain intensity, physical capacity, stress, and depression. 40% improved with clinical important change in self-reported activity related to CLBP over a period of 13-years, which is important information to provide to individuals seeking primary healthcare for CLBP.

**Trial registration:**

ClinicalTrials.gov, ID: NCT03974191. Registration date: 27 May 2019.

## Background

Low back pain (LBP) is a highly prevalent musculoskeletal health problem [1], which causes more global disability than any other condition [2, 3] and is a common reason for seeking primary healthcare [4, 5]. Musculoskeletal disorders are among the main causes of long-term sickness absence and exclusion from the labor market due to LBP and is associated with considerable costs for both the individual and society [4, 6].

The course of acute LBP is often described as recovery or with significant reductions in pain and disability levels within 6 weeks [5, 7]. However, at one year follow-up, 63% to 82% continue to have LBP, and 20% to 45% report functional and activity limitations [8–10]. The clinical presentation of chronic LBP (CLBP) is defined as LBP with a duration longer than 12 weeks and is associated with variations of recurrent or persistent pain altered with pain free periods with varying impact on body function, activity, and participation in daily living [11]. CLBP is multi-factorial and can be caused by a combination of physical, environmental, lifestyle-related, and psychological factors, which can impact the ability to perform daily activities, fulfill social roles and participate in work life [12, 13]. Recently, a meta-analysis investigating the clinical course of acute, sub-acute and CLBP suggests that additional knowledge is needed to enhance the certainty of evidence on the clinical course of CLBP over an extended period of time [7].

Various prognostic factors have been identified for the improvement of CLBP such as; physical activity/exercise [14], but physical activity in relation to CLBP is suggested to be U-shaped i.e. both insufficient and excessive physical activity can be equally harmful to spinal health [15]. Persistent multisite pain, poor health, fear avoidance, pain-related disability and psychological components have been shown to reduce the probability to recover [16–19]. Additionally, physical capacity i.e. the 6-Minute Walk Test (6MWT) and symptoms of depression have been shown to predict the probability of future work ability in women with CLBP [20].

Findings from a long-term prospective longitudinal cohort study [20] can provide guidance for interventional trials aiming to improve function, activity, participation in daily life and other health outcomes, which also might decrease the risk for developing chronic diseases for this large and costly population [3, 6]. The knowledge about the clinical course of CLBP for improvement in body function, activity and participation and the recovery of CLBP over a period of over ten years is still insufficient [7, 21]. Pain severity can be viewed as a continuum, ranging from no pain to regional pain to widespread pain (WP), with various intermediate levels. Individuals have been observed to transition between different severities of pain in both directions over time [22]. Long-term monitoring might provide a better understanding about the characterization of pain and disability progression over time, and the identification of patterns that may be instrumental in understanding the progression of CLBP. CLBP and WP have been shown to be more prevalent among women, and associated with more pain severity [1]. Nevertheless, sex-specific analyses remain limited [23]. In a cohort of women with CLBP, lower physical capacity, more severe clinical stress symptoms, and greater activity limitation are associated with increased activity limitation over time [12]. Additionally, the presence of WP is associated with more impaired physical capacity, more severe pain and fatigue, and more severe symptoms of depression and clinical stress compared to those with only CLBP [13]. Over a period of more than ten years, women with both CLBP and WP might exhibit a different clinical course of self-reported activity limitations compared to women with only CLBP, taking baseline characteristics (e.g. age, education, pain intensity, physical capacity, symptoms of stress, and depression) and individual variability into account.

This 13-year longitudinal study follows a cohort of women with CLBP seeking primary healthcare [12, 13], and investigates whether the long-term development of self-reported activity e.g. Roland Morris Disability Questionnaire (RMDQ) [24, 25] differs between women with CLBP alone and those with both CLBP and WP. In addition, the long-term change for body function, self-reported activity and, participation in work were investigated. The rationale for this study is to address the current knowledge gap regarding the long-term clinical course of CLBP and its impact on body function, activity, and participation. By comparing women with CLBP alone to those with both CLBP and WP over a 13-year period, this study provides insight into trajectories of disability. Identifying these patterns has important clinical utility for improving rehabilitation strategies, sustaining work ability, and preventing further health deterioration.

## Methods

### Aim

The aim of the study was to investigate the long-term change in body function, self-reported activity e.g. Roland Morris Disability Questionnaire (RMDQ) and work participation in women with CLBP. Additionally, the study aimed to investigate whether the long-term development of self-reported activity differs between women with CLBP only and those with both CLBP and WP. We hypothesise that the cohort as a whole will show small improvements in body function, self-reported activity and work participation over time, based on evidence of improvements observed in other chronic pain conditions [26]. Furthermore, we hypothesise that women with both CLBP and WP will report more severe disability after 13 years than women with only CLBP.

### Study design

This is a 13-year prospective longitudinal cohort study of women with CLBP seeking primary healthcare. The original recruitment procedure was conducted in 2004–2005 across eight primary healthcare centers in Southwestern Sweden. Through a systematic review of medical records for ICD-10 LBP diagnosis (M545), female patients were identified. Those who could be contacted, consented to participate, and met the inclusion criteria were invited to enroll in the study. The participants were assessed in 2004 to 2005 [13], after two years [12, 20] and after 13 years. Because of the pandemic of COVID-19 and the associated restrictions, the recruitment to the 13-year follow-up was cancelled in spring 2020.

The inclusion criteria for the first assessment in 2004 to 2005 [13] were as follows: female and LBP (pain between costal margins and gluteal folds) with or without referred leg pain [5], symptom duration of more than 12 weeks, not pregnant, no known spinal disorders (infection, tumor, fracture, inflammatory disease, spinal stenosis), no other severe psychiatric or somatic disorders, age between 18 and 60 years, and understanding and being proficient in Swedish. At the 13-year follow-up, all participants (*n* = 130) included in the first assessment [13], who could be contacted and accepted participation were asked to participate in the 13-year follow-up. The 13-year follow-up adhered to the same study protocol as the first assessment and the two-year follow-up. Written informed consent was obtained from all participants.

### Procedure

The participants were assessed by physical therapists at primary healthcare rehabilitation clinics who were trained in the assessment protocol. The same physiotherapists conducted the examinations at baseline, at the two-year follow-up, and at the 13-year follow-up. The assessment included standard questions about socio-demographic data, history of LBP, comorbidity, and pharmacological treatment as well as physical performance tests. Additionally, the participants were asked to complete patient reported outcome questionnaires.

### Measurements

#### Body function

##### Physical performance tests

A walk test, 6MWT was used to measure physical capacity [27] considered reliable and valid for patients with chronic pain [28]. The Grippit was used to measure voluntary hand grip force [29].

##### Pain assessment

The localisation and distribution of pain for more than three months during the last 12 months were reported in a self-administered pain drawing with predefined body regions, referring to the number of body regions in pain [1, 30].

Pain intensity during the previous week was registered using a Visual Analogue Scale (VAS) ranging from 0 to 100 mm, with a higher score indicating more severe pain [31]. A moderately clinically important improvement has been suggested to be ≥ 30% [32, 33]. The impact of pain on daily life was registered with VAS (0 to 100 mm), with a higher score indicating more impact on daily life.

##### Distress

The Hospital Anxiety Depression Scale (HADS) was used for anxiety and depression [34]. The scores build two subscales for anxiety (HADS-A) (0 to 21 points) and depression (HADS-D) (0 to 21 points). A higher score indicates more severe symptoms.

### Activity related to CLBP

For self-reported activity related to CLBP, the Roland Morris Disability Questionnaire (RMDQ) [24] was used. Higher scores indicate more severe activity limitation (0 to 24 points) [24]. In this study, a one-point change in either direction was used as an indicator of improvement or deterioration. The minimal important clinical change is commonly considered to be 4 to 5 points [32, 35].

### Other health-related aspects

The Stress and Crisis Inventory-93 (SCI-93) assesses 35 clinical manifestations of stress, encompassing both physical and mental sensations. Total score is ranging from 0 to 140 points. A higher score indicates more clinical stress symptoms [36].

Short Form-36 (SF-36) assesses health-related quality of life through eight subscales, each ranging from 0 to 100. These subscales build two composite scores, the Physical Component, and the Mental Component. A higher score indicates better health-related quality of life [37, 38].

### Study sample

Our aim for the first assessment at baseline in 2004 to 2005 was to include just over 100 participants who had sought primary healthcare over a one-year period, to investigate changes in body function, activity, participation, and to reasonably identify prognostic factors regarding self-reported activity ability over time. A previous cohort study, conducted in primary healthcare followed 78 individuals with LBP and achieved a response rate of 70% (47/78) after two decades [39]. We were anticipating that the response rate would reach similar levels. In our study, the cohort comprised 131 women with CLBP at the first assessment.

### Data analysis

Group characteristics at baseline were analysed descriptively and are presented as mean and standard deviations, median and 25th and 75th percentile or the number and percentage. Measurements were analysed by calculating raw differences between first assessment and the 13-year follow-up. Parametric and non-parametric tests were used depending on data level and distribution.

To compare the differences between the group that was followed up and the group that was lost to follow-up after 13 years, T-test was used for continuous data such as pain duration, and the Mann-Whitney U-test was used for ordinal data or skewed distributions. For categorical data, Chi-square tests and Fisher’s exact tests were employed. To compare change between the first assessment and the 13-year follow-up, McNemar test for binary variables, Wilcoxon Rank Test for ordinal data and paired sample t-test for continuous data was used. The two-year follow-up data were not analysed as a separate outcome but included in the mixed effect model analysis, with RMDQ as the outcome measure, as described below.

At baseline, pain locations [1] were used to categorise the participants into two groups; localised CLBP or CLBP and fulfilling the American College of Rheumatology (ACR) 1990 criteria for widespread pain (WP) [40] (CLBP + WP). Education was collected in three categories. For the statistical analysis, the variable was dichotomised; up to 12 years or >12 years, due to the small number of participants in one category.

A mixed effect model analysis was performed for repeated measures of activity (RMDQ) from baseline to two-year and 13-year follow-up comparing the group localised CLBP and CLBP + WP. The RMDQ was the dependent variable. Independent variables were age, education level, pain intensity (VAS), physical capacity (6MWT), clinical symptoms of stress (SCI-93) and symptoms of depression (HADS-D). Body Mass Index (BMI) was not included as a confounding factor, as it did not predict RMDQ outcomes at the two-year follow-up [12]. Before the mixed effect model analysis, assumptions were checked using Spearman’s rank correlation coefficient (*r* ≤ 0.7), overlapping boxplot, and cross tables (> 80% observations in diagonal and cells < 5 observations) for possible collinearity. The mixed effect model analyses were performed analysing Time, Group (CLBP or CLBP + WP) and the interaction between Time and Group. Model 1 included the variable Time, Group (CLBP or CLBP + WP), Time x Group and one confounder added one at a time (age and education) and then with possible secondary confounders added one at a time (pain intensity (VAS), 6MWT, SCI-93 and HADS-D). Model 2, the final model, comprised Model 1 with confounders from Model 1 *p* < 0.20, Time, Group, Time x Group, pain intensity (VAS), 6MWT, SCI-93 and HADS-D. To illustrate the change in RMDQ for the groups CLBP and CLBP + WP (Model 2, final model) over a period of 13 years, the estimated model means for RMDQ were used and presented in graphs. Finally, a sensitivity analysis was conducted and included only participants who could be followed up at the 13-year follow-up.

Missing data were addressed in accordance with the guidelines provided in the respective manuals. For the SCI-93 and HADS scales, isolated missing responses were imputed by calculating the mean of the completed items within the relevant subscale. For SCI-93 a maximum of four missing responses are permissible. In such cases, the missing values were imputed using the mean of the respondent’s available item scores. Subsequently, a total score was computed based on the imputed dataset. Regarding the RMDQ, if a participant had marked both “yes” and “no” for a single item, the response was interpreted as “yes.” In cases where an item was left unanswered, it was treated as a “no” response.

The level of significance was set at *p* < 0.05. The IBM SPSS Windows version 25 was used for all statistical analyses. The report follows the STROBE statement.

## Results

At the 13-year follow-up, most outcome measures indicated either minor improvements or remained stable. No significant differences were observed in RMDQ between the groups CLBP and CBLP + WP, adjusting for baseline pain intensity, physical capacity, symptoms of stress, and depression.

**Fig. 1:**
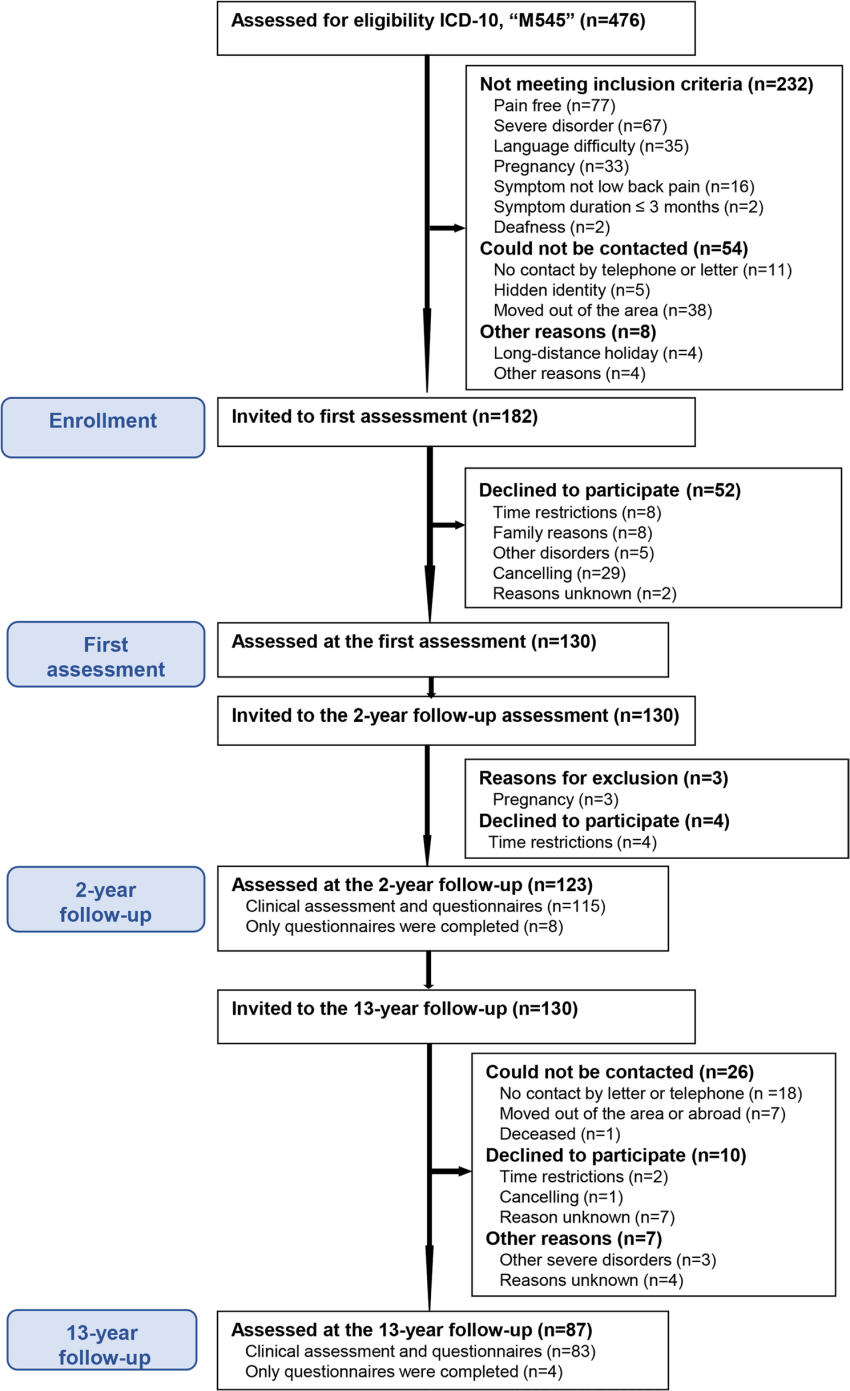
The participants flow

67% (87/130) could be followed up after 13 years (Fig. 1). For the group that was not followed-up at 13 years (*n* = 44), no difference was observed in personal and socio-demographic factors except Swedish nationality (93% (80/86) vs. 77% (34/44), *p* = 0.021) (Table 1). Mental health was lower at the first assessment in the group that was not followed up (Table 1). Thirty-one participants (36%) had retired since first assessment and were not included in the analysis of work ability (Table 1). 26% of the participants (22/86) fulfilled the ACR 1990 criteria for WP [40] at baseline. The estimated sample distributions, separately for the groups CLBP and CLBP + WP, are presented in Table 2.

**Table 1:** Participant characteristics at first assessment in 2004–2005 for the women who were followed up or not after 13 years and the p-values for the difference between the groups

	Followed up after 13 years (*n* = 87)	Not followed up (*n* = 44)	*p*-values
Personal and socio-demographic factors	Mean (SD) --- median (25th; 75th percentile) or percent (n/n total)		
Age-years	46 (11) --- 49 (37;54)	43 (9.3) --- 43 (37;51)	0.13
Nationality-Swedish [%(n/n)]	93% (80/86)	77% (34/44)	0.021
Symptom duration - years	9.8 (8.9) --- 6.0 (2.0;15)	8.9 (8.3) --- 5.5 (3.0;12)	0.62
Pharmacological treatment [%(n/n)]^a^			
Analgesics	49% (42/86)	64% (28/44)	0.14
Psychotropic drugs	12% (10/86)	25% (11/44)	0.076
Education [%(n/n)]			
≤ 9 years	13% (11/86)	11% (5/44)	0.58
10–12 years	36% (31/86)	46% (20/44)	
> 12 years	51% (44/86)	43% (19/44)	
Social status [%(n/n)]			
Living with an adult	32% (27/85)	14% (6/44)	0.23
Living with an adult and child/children	45% (38/85)	59% (26/44)	
Living alone	12% (10/85)	11% (5/44)	
Living alone with child/children	7.1% (6/85)	11% (5/44)	
Living apart with an adult	4.7% (4/85)	4.5% (2/44)	
Body function			
Pain intensity (VAS 0–100 *mm*)	41 (25) --- 35 (23;62)	54 (29) --- 52 (28;75)	0.013
Pain localisations (0–18 *sites*)	4.4 (2.8) --- 4 (2.0;6.0)	5.0 (3.8) --- 4 (2.0;7.0)	0.52
6-Minute Walk Test, *meter*	574 (82) --- 580 (527;633)	570 (94) --- 585 (514;633)	0.94
Hand grip force, *Newton*^b^	236 (67) --- 238 (200;278)	221 (89) --- 226 (153;290)	0.49
Anxiety (HADS-A) (0–21 *p)*^c^	5.6 (3.9) --- 5.0 (3.0;8.0)	8.0 (5.1) --- 7.5 (4.0;12)	0.0077
Depression (HADS-D) (0–21 *p*)^c^	3.7 (3.1) --- 3.0 (1.0;5.0)	5.7 (4.2) --- 6.0 (2.0;7.8)	0.012
Activity			
Roland Morris Disability Questionnaire (0–24 *p*)	7.9 (4.3) --- 7.0 (4.8;10)	10 (5.8) --- 11 (4.0;15)	0.065
Participation			
Employment Status [%(n/n)]			
Available for work	84% (72/86)	68% (30/44)	0.070
Not available for work	16% (14/86)	32% (14/44)	
Other health-related factors			
SCI-93 (0–140 *p*)^d^	36 (21) --- 35 (19;51)	38 (22) --- 37 (19;53)	0.63
SF-36 PCS (0–100 *p*)^e^	39 (9.5) --- 40 (32;47)	36 (11) --- 38 (28;44)	0.11
SF-36 MCS (0–100 *p*)^e^	48 (12) --- 52 (40;57)	42 (14) --- 40 (32;53)	0.011

^a^Analgesic and psychotropic pharmacies used last month (yes)

^b^Right hand 10 s

^c^Hospital Anxiety Depression Scale. A higher score indicates more severe symptoms

^d^Stress and Crisis Inventory-93 (SCI-93). A higher score indicates more severe stress symptoms

^e^Short Form-36, the Physical Component Score (PCS),Mental Component Score (MCS). A higher score indicates better health-related quality of life

**Table 2:** Participant characteristics at first assessment in 2004–2005 in the group with localised chronic low back pain (CLBP) and the group CLBP plus widespread pain (WP)* who could be followed up after 13 years

	CLBP (*n* = 64)	CLBP + WP (*n* = 22)
Personal and socio-demographic factors	Mean (SD) --- median (25th; 75th percentile) or percent (n/n total)	
Age-years	45 (11) --- 47 (37;54)	48 (11) --- 52 (40;56)
Nationality-Swedish [%(n/n)]	92% (59/64)	96% (21/22)
Symptom duration - years	9.7 (9.0) --- 6.0 (2.0;15)	9.9 (8.6) --- 7.0 (4.4;14)
Pharmacological treatment [%(n/n)]^a^		
Analgesics	42% (27/64)	68% (15/22)
Psychotropic drugs	7.8% (5/64)	23% (5/22)
Education [%(n/n)]		
≤ 9 years	9.4% (6/64)	23% (5/22)
10–12 years	34% (22/64)	41% (9/22)
> 12 years	56% (36/64)	36% (8/22)
Social status [%(n/n)]		
Living with an adult	30% (19/63)	36% (8/22)
Living with an adult and child/children	48% (30/63)	36% (8/22)
Living alone	9.5% (6/63)	18% (4/22)
Living alone with child/children	6.3% (4/63)	9.1% (2/22)
Living apart with an adult	6.3% (4/63)	0% (0/22)
Body function		
Pain intensity (VAS 0–100 *mm*)	37 (23) --- 28 (20;57)	53 (28) --- 53 (28;70)
Pain localisations (0–18 *sites*)	3.1 (1.6) --- 3.0 (2.0;4.0)	8.0 (2.4) --- 8.0 (6.0;10)
6-Minute Walk Test, *meter*	593 (78) --- 601 (558;651)	520 (66) --- 529 (464;577)
Hand grip force, *Newton*^b^	246 (66) --- 245 (212;284)	208 (61) --- 202 (159;269)
Anxiety (HADS-A) (0–21 *p)*^c^	5.8 (3.9) --- 5.0 (3.0;8.0)	4.9 (4.0) --- 4.0 (2.0;6.0)
Depression (HADS-D) (0–21 *p*)^c^	3.5 (3.0) --- 3.0 (1.0;5.0)	4.1 (3.4) --- 3.0 (1.0;6.0)
Activity		
Roland Morris Disability Questionnaire (0–24 *p*)	7.3 (4.3) --- 7.0 (4.0;9.0)	9.7 (3.8) --- 9.5 (7.0;13.0)
Participation		
Employment status [%(n/n)]		
Available for work	86% (55/64)	77% (17/22)
Not available for work	14% (9/64)	23% (5/22)
Other health-related factors		
SCI-93 (0–140 *p*)^d^	32 (20) --- 27 (13;43)	48 (21) --- 44 (28;60)
SF-36 PCS (0–100 *p*)^e^	40 (9.6) --- 42 (34;48)	36 (8.7) --- 36 (29;42)
SF-36 MCS (0–100 *p*)^e^	48 (12) --- 52 (39;57)	47 (14) --- 52 (42;56)

^*^Localised chronic low back pain (CLBP) or having CLBP and fulfilling the American College Rheumatology 1990 criteria for widespread pain [40] (CLBP + WP)

^a^Analgesic and psychotropic pharmacies used last month (yes)

^b^Right hand 10 s

^c^Hospital Anxiety Depression Scale. A higher score indicates more severe symptoms

^d^Stress and Crisis Inventory-93 (SCI-93). A higher score indicates more severe stress symptoms

^e^Short Form-36, the Physical Component Score (PCS), Mental Component Score (MCS). A higher score indicates better healthrelated quality of life

### Change between first assessment and 13-year follow-up

All measurements but hand grip strength improved or were stable (Table 3). Both pain intensity (−9.7 (sd 28) mm) and how pain impacts daily life (−11 (sd 31) mm) showed a statistically significant decrease (*p* < 0.05) after 13 years, while the number of pain locations remained stable. A total of 41 women (48%) showed a moderately clinically important improvement in pain intensity (≥ 30% improvement from baseline) [32, 33] after 13 years. Anxiety (HADS-A) (−0.91 (sd 4.2) points) and clinical symptoms of stress (SCI-93) (−3.0 (sd 16) points) also showed a statistically significant decrease (*p* < 0.05). Physical health-related quality of life (SF-36 PCS) significantly improved (4.6 (sd 10) points, *p* < 0.001) whereas mental health-related quality of life (SF-36 MCS) remained stable (Table 3). In RMDQ, 73% (63/86) improved with one point of change in self-reported activity related to PBLP (*p* < 0.001) (Table 3). Specifically, 40% (34/86) improved by ≥ 5 points and 34% (29/86) by 1–4 points, while 10% (9/86) were unchanged (0 points) and 16% (14/86) worsened by ≥ 1 point. At the 13-year follow-up, work ability remained unchanged among those who were still occupationally active (Table 3).

**Table 3:** Change between first assessment and the 13-year follow-up (*n* = 87)

Body function	First assessment*	13-year follow-up*	Change*	*p*-values**	Improved % (*n*/*n*)
Body Mass Index (BMI) (*n* = 81)	27 (5.3) 26 (23;31)	27 (4.9) 26 (24;30)	0.72 (3.5) 0.90 (−0.6.0;2.8)	**0.0048**	33% (26/79)
Pain intensity (VAS 0 to 100 *mm*)	41 (25) 34 (23;62)	32 (25) 23 (11;49)	−9.7 (28) −9.5 (−30;11)	**0.0063**	63% (54/86)
How pain impacts daily life (VAS 0 to 100 *mm*)	46 (23) 46 (26;66)	35 (29) 30 (9.0;59)	−11 (31) −10 (−33;6.0)	**0.0011**	67% (57/85)
Pain localisations (0 to 18 *sites*)	4.4 (2.8) 4.0 (2.0;6.0)	4.6 (3.9) 4.0 (2.0;5.0)	0.12 (3.6) 0.0 (−2.0;2.0)	0.90	43% (37/86)
6-Minute Walk Test, *meter* (*n* = 79)	574 (82) 580 (527;633)	551 (104) 556 (486;624)	−19 (86) −9.0 (−85;32)	0.057	42% (32/77)
Hand grip force, *Newton*^*a*^ (*n* = 81)	236 (67) 238 (200;278)	195 (73) 186 (146;255)	−41 (54) −46 (−82; −3.1)	**< 0.001**	21% (17/80)
Anxiety (HADS-A, 0–21 *p*)^b^	5.6 (3.9) 5.0 (3.0;8.0)	4.7 (3.9) 3.0 (2.0;7.0)	−0.91 (4.2) −1.0 (−3.0;1.0)	**0.015**	55% (47/86)
Depression (HADS-D, 0–21 *p*)^b^	3.7 (3.1) 3.0 (1.0;5.0)	3.1 (3.1) 2.0 (1.0;4.0)	−0.57 (3.4) 0.0 (−3.0;1.0)	0.17	48% (41/86)
Activity					
Roland Morris Disability Questionnaire (0 to 24 *p*)	7.8 (4.3) 7.0 (4.8;10)	4.6 (4.6) 3.0 (1.0;8.0)	−3.3 (4.1) −3.0 (−6.0;0.0)	**< 0.001**	73% (63/86)
Participation					
Employment status [% (n/n)]					
Available for work	84% (72/86)	58% (50/87)		0.34***	
Not available for work	16% (14/86)	6.9% (6/87)			
Retired	N/A	36% (31/87)			
Other health-related factors					
SCI-93 (0 to 140 *p*)^c^	36 (21) 35 (19;51)	33 (25) 27 (13;46)	−3.0 (16) −4.0 (−13;5.0)	**0.026**	63% (54/86)
SF36-PCS (0 to 100 *p*)^d^	39 (9.5) 40 (32;47)	43 (10) 46 (37;51)	4.6 (10) 4.4 (−2.4;10)	**< 0.001**	67% (58/86)
SF36-MCS (0 to 100 *p*)^d^	48 (12) 52 (40;57)	49 (13) 53 (42;58)	0.61 (14) 0.83 (−7.0;9.0)	0.41	51% (44/86)

*Mean value (standard deviation), median value (25th;75th percentile) for change between first assessment and 13 years

**Wilcoxon Signed Rank Test for change between first assessment and 13-year follow-up

***McNemar Test. Retired participants are not included in the analysis (valid cases for the analysis *n* = 55)

^a^Right hand 10 s ^b^Hospital Anxiety Depression Scale. A higher score indicates more severe symptoms

^c^Stress and Crisis Inventory-93 (SCI-93). A higher score indicates more severe stress symptoms

^d^Short Form 36, the Physical Component Score (PCS), Mental Component Score (MCS). A higher score indicates better health-related quality of life

### Improvement in self-reported activity related to CLBP after 13 years adjusting for confounders

**Fig. 2:**
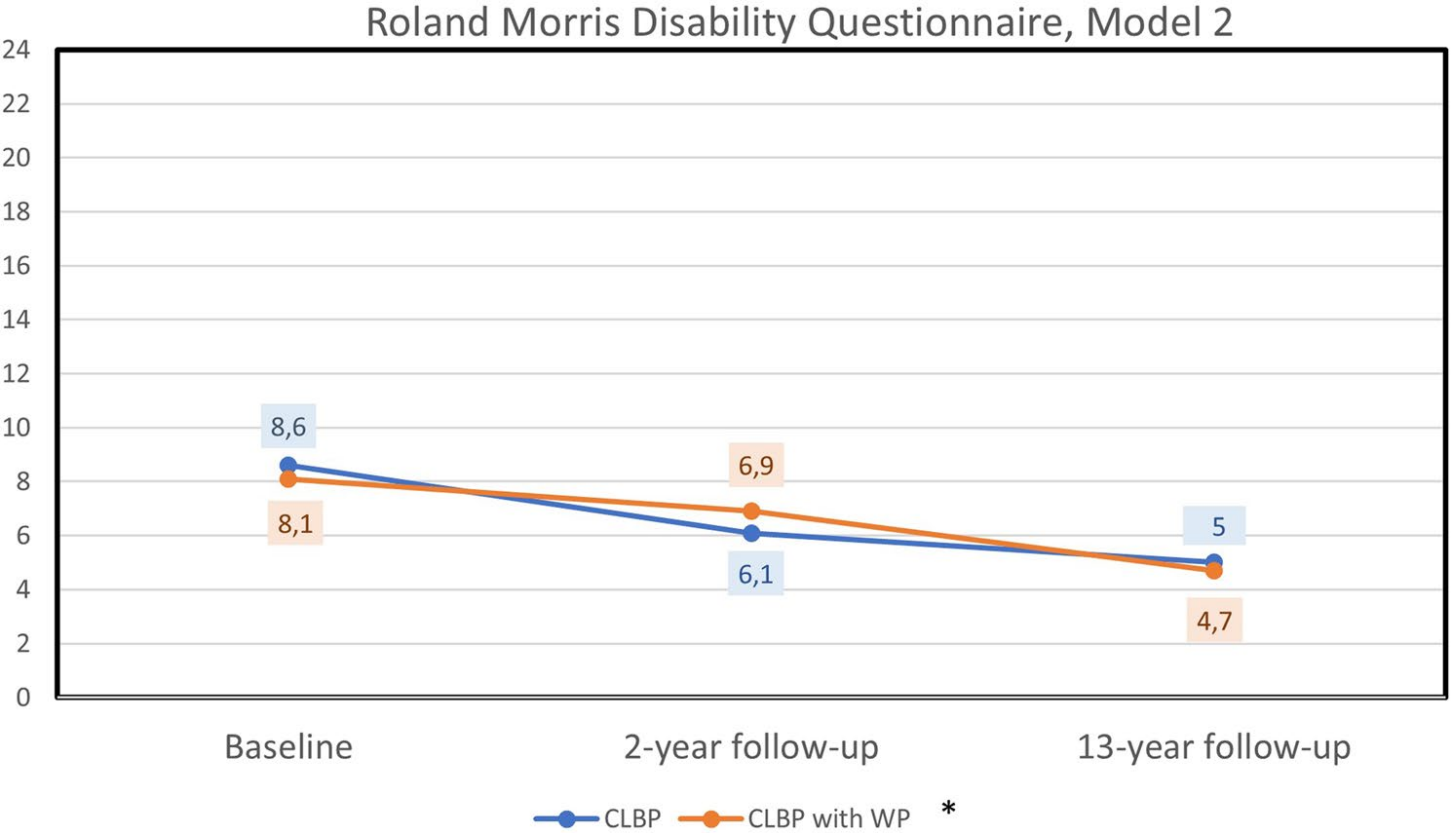
Repeated measures of self-reported activity (RMDQ) from baseline to two-year and 13-year follow-up for the group with localised chronic low back pain (CLBP) and the group with CLBP and widespread pain (WP) Estimated marginals means for Roland Morris Disability Questionnaire (0 to 24 points). Higher scores indicate more severe disability due to low back pain. Model 2 adjusted for possible confounders (Pain intensity last week, 6 Minute Walk Test, Stress and Crises Inventory – 93 (0 to 140 points) and Hospital Anxiety and Depression Scale – Depression (0 to 21 points). Covariates appearing in the model are evaluated at the following values: Pain intensity at baseline (VAS 0 to 100 mm) = 44.0060, 6-Minute Walk Test at baseline (meter) = 572.7798, Stress and Crisis Inventory- 93 at baseline (0 to 140 points) = 35.9601, Depression HADS at baseline (0 to 21 points) = 4.1848 *Localised Chronic Low Back Pain (CLBP) or having CLBP and fulfilling the American College Rheumatology 1990 criteria for Widespread Pain (WP)

Self-reported activity related to CLBP (RMDQ) improved in both groups (CLBP or CLBP + WP) over the 13-year period (*p* < 0.001) when controlling for age, education level, pain intensity, 6MWT, SCI-93 and HADS-D (Table 4). The estimated model means for RMDQ were 8.6 points at baseline, 6.1 points after two years, and 5.0 points after 13 years for the group with localised CLBP and for the group with CLBP + WP the estimated means were 8.1 points at baseline, 6.9 points after at two years, and 4.7 points after 13 years (Fig. 2). The results from Model 2 remained consistent in the sensitivity analysis, which included only the participants who could be followed up at 13 years (Table 4). For the participants who could be followed up after 13-years, the estimated model means for RMDQ were 8.1 points at baseline, 6.0 points after two years, and 4.7 points after 13 years for the group with localised CLBP and for the group with CLBP + WP the estimated means were 7.4 points at baseline, 5.3 points after at two years, and 4.3 points after 13 years.

**Table 4:** Mixed effect model analysis for repeated measures for self-reported activity due to chronic low back pain, Roland Morris disability Questionnaire, from baseline to 2-years and 13-year follow-up

Model 1		Model 1 with possible confounders						Model 2^g^		Sensitivity analysis^h^
*Variables*	*p-values*	*p-values*						*Estimates of* *Fixed Effects* *(95%CI)*	*p-values*	*p-values*
Time	< 0.001	<0.001	<0.001	< 0.001	< 0.001	< 0.001	< 0.001	---	<0.001	<0.001
Baseline								3.4 (1.7–5.1)	<0.001	
2-years								2.1 (0.23–4.0)	0.028	
13-years								**	**	
Group (CLBP (1) CLBP + WP (2))^a^	< 0.001	< 0.001	< 0.001	0.0042	0.022	0.052	< 0.001	0.22 (−1.7−2.2)*	0.82	0.46
								**	**	
Time x Group	0.34	0.34	0.34	0.35	0.34	0.35	0.37	---	0.38	0.96
Baseline x Group CLBP								0.20 (−1.8−2.2)	0.84	
2-years x Group CLBP								−0.95 (−3.2−1.3)	0.40	
13-years x Group CLBP								**	**	
Possible confounders at baseline, fixed data^b^		*P*-values for confounders when added separately to the Model 1								
Age, *year*		0.38								
Education^c^			0.38							
Possible secondary confounders at baseline, fixed data^b^		*P*-values for secondary confounders when added separately to the Model 1								
Pain intensity last week, (VAS, 0–100 *mm*)^d^				< 0.001				0.030 (0.0075–0.052)	0.0090	0.025
6-Minute Walk Test (*meter)*					< 0.001			−0.021 (−0.028- −0.014)	<0.001	<0.001
SCI-93 (0 to 140 *p*)^e^						< 0.001		0.070 (0.035–0.11)	<0.001	<0.001
Depression, HADS-D (0 to 21 *p*)^f^							< 0.001	0.18 (−0.016−0.38)	0.071	0.38

*Estimates for Group CLBP

**Reference

^a^Localised chronic low back pain (CLBP) or having CLBP and fulfilling the American College Rheumatology 1990 criteria for widespread pain [40] (CLBP + WP)

^b^Confounders with fixed baseline data were added separately to the Model 1

^c^Education dichotomized in two groups: up to 12 years (1), >12 years (2)

^d^VAS, Visual Analogue Scale

^e^SCI-93, Stress and Crisis inventory-93, higher scores indicate more severe clinical symptoms of stress

^f^ Hospital Anxiety and Depression Scale, subscale for depression. Higher scores indicate more severe symptoms of depression

^g^Confounders added one at the time to Model 1, with p-values < 0.2 were included in Model 2 (Time, Group, Time x Group, pain intensity, 6-Minute Walk Test, SCI-93, HADS-D)

^h^Sensitivity analysis including only the participants who could be followed up at 13-years

## Discussion

At the 13-year follow-up, all outcome measurements showed small improvement or stability, except for hand grip force. One fourth of the women fulfilled the ACR 1990 criteria of WP at baseline. Both groups, CLBP and CLBP + WP, improved in self-reported activity related to CLBP (RMDQ) and there was no statistically significant difference in RMDQ, when adjusted for pain intensity, 6MWT, SCI-93 and HADS-D, between the two groups after 13 years. The participants improved in self-reported activity related to CLBP (RMDQ) over a period of 13 years, with 40% showing an improvement of at least 5 points, reflecting important clinical change [32, 35], which is important information to give patients seeking primary healthcare for CLBP.

In this study of women with PLBP in primary care, the estimated mean value showed moderate to low levels (< 14 points) of activity limitation (RMDQ) at baseline, two and 13-year follow-up (Fig. 2), suggesting that a small number of women in this cohort had severe (≥ 14 points) activity limitation due to CLBP, which is concordant to a study in secondary care [21]. It should be emphasised that low to moderate levels of activity limitation, as measured with RMDQ, can be associated with considerable suffering over time even if improvements are shown. A recently published meta-analysis, indicates that individuals with CLBP continue to experience moderate-to-high levels of pain and disability after one year and they discuss the need to develop more effective treatment for individuals with CLBP [7].

Physical capacity has been shown to be a predictor for improvement in CLBP [14], activity [12] and work ability [20]. In this study, the mean value for the 6MWT was 574 (sd 82) meters at the first assessment, which can be compared to a mean value of 581 (sd 67) meters from studies of healthy individuals in the general population [41]. It is encouraging that the participants have maintained good walking capacity after 13 years, with a mean value of 551 m (sd 104). Given that the group started with such good walking capacity, further improvement on this test was not anticipated. Hand grip force significantly declined between baseline and 13 years, which could be expected, as grip force decreases with age [29].

CLBP often has a complex background [3] which is why rehabilitation comprising a bio-psycho-social treatment may be composed of various interventions offered from different professionals [4]. However, intensive, supervised, clinic-based treatment for long periods requires commitment on behalf of the individual and is costly. It has been suggested that CLBP can be managed with effective, safe and low-cost interventions such as self-management programs [42] and self-managing interventions based on a cognitive behavioural therapy framework have shown moderate effect on pain intensity and disability [43]. Against this background, the present long-term cohort study can provide insights into changes in recommended outcomes for women with CLBP over a period of 13 years. These data can contribute to the understanding of the trajectory and might facilitate the development of interventions. Moreover, in this study, the same study protocol was used at all assessments including measurements across all domains in the International Classification of Functioning, Disability and Health (ICF) [44], providing a comprehensive overview of the participants. The use of knowledge how CLBP improves over a long period of time, when planning and interacting with an intervention might better improve the prognosis [45], but needs to be studied further.

### Strengths and limitations

CLBP is multifactorial and a set of measurements, such as physical functioning, pain intensity and health-related quality of life, in research have been recommended [46]. In this study, the same study protocol was used at all assessments incorporating measurements across all domains of the ICF encompassing body function, activity, participation and health-related quality of life, which is in line with the recommendations [46]. Restricting the study only to women with CLBP limits the generalisability to men. However, although both CLBP and WP are more prevalent among women [1], this study did not aim to investigate sex differences. Consequently, only female participants were included.

67% (87/130) of the women were successfully followed up after 13 years, which can be considered a strength. It is important to consider that those lost to follow-up after 13 years included a lower percentage of individuals of Swedish nationality and exhibited significantly poorer mental health, which may affect the interpretation of the results. A sensitivity analysis (Table 4) was conducted to determine if the missing outcomes from participants who could not be followed up affected the results for change in RMDQ. The analysis showed that this was not the case.

Participants were categorised into having CLBP or CLBP + WP based on predefined criteria, resulting in unequal group sizes. This imbalance does not imply bias, as the mixed effects model used accommodates unbalanced data and accounts for group-level variability and intra-group dependencies, enhancing estimate validity. A formal adjustment for multiple testing was not done in the analysis of the change between first assessment and the 13-year follow-up. Hence, this should be considered in the interpretation. In the analysis of the RMDQ, using a mixed effects linear model, no adjustments were done as only the p-values for the primary variables are interpreted, not the p-values related to the confounders.

The transition from acute to CLBP is influenced by prognostic factors, which are typically negative in patients who develop chronic pain. In this study, we did not focus on prognostic factors for self-reported activity limitation (RMDQ) after 13 years. Instead, the aim was to describe long-term disability trajectories in women with CLBP or CLBP + WP. The mixed effect model analysis was adjusted for baseline variables including age, educational level, pain intensity, physical capacity, stress-related clinical symptoms, and symptoms of depression.

There are a lot of factors that can influence self-reported activity limitation in CLBP. Treatment was not controlled for in this study, as it was not the primary aim, and the inclusion criteria ensured a relatively homogeneous population. Although treatment was not the focus, its potential impact on trajectories warrants consideration. It is likely that participants received various interventions over time, which may have influenced the course and outcomes. This should be considered when interpreting the findings. Confounders were selected based on known factors to be associated with self-reported function, activity and, participation [12, 20, 47, 48]. Moreover, we performed mixed effect model analyses, which are useful when repeated measures on the same individual are available [49]. This model provide insight into how the baseline assessment of the women with CBLP or CLBP + WP influenced their self-reported activity (RMDQ) at the 13-year follow-up, adjusting for potential confounders. In this mixed effect model analysis, baseline pain intensity, 6MWT, SCI-93 and HADS-D were adjusted, which used each woman´s individual change.

## Conclusion

This study showed that all outcome measurements except for hand grip force either showed small improvements or remained stable. We found no difference in self-reported activity related to CLBP at the 13- year follow-up, adjusted for pain intensity, physical capacity, stress, and depression for the group CLBP compared to the group CLBP with WP. The participants improved in self-reported activity related to CLBP over a period of 13 years which is important information to provide individuals seeking primary care for CLBP.

## Data Availability

The datasets used and/or analysed during the current study are available from the corresponding author (LN) on reasonable request.

## Abbreviations

ACR: American college of rheumatology
CLBP: Chronic low back pain
HADS-A: Hospital anxiety depression scale - anxiety
HADS-D: Hospital anxiety depression scale - depression
ICF: International classification of functioning, disability and health
LBP: Low back pain
MCS: Mental component score
PCS: Physical component score
RMDQ: Roland morris disability questionnaire
SCI-93: Stress and crisis inventory-93
SF-36: Short form-36
VAS: Visual analogue scale
WP: Widespread pain
6MWT: 6-Minute walk test
